# Metabolic rewiring and redox alterations in malignant pleural mesothelioma

**DOI:** 10.1038/s41416-019-0661-9

**Published:** 2019-12-10

**Authors:** Loredana Urso, Ilaria Cavallari, Evgeniya Sharova, Francesco Ciccarese, Giulia Pasello, Vincenzo Ciminale

**Affiliations:** 1 Department of Surgery, Oncology and Gastroenterology, University of Padua, Padua, Italy; 2 Veneto Institute of Oncology IOV – IRCCS, Padua, Italy

**Keywords:** Mesothelioma, Cancer metabolism

## Abstract

Malignant pleural mesothelioma (MPM) is a rare malignancy of mesothelial cells with increasing incidence, and in many cases, dismal prognosis due to its aggressiveness and lack of effective therapies. Environmental and occupational exposure to asbestos is considered the main aetiological factor for MPM. Inhaled asbestos fibres accumulate in the lungs and induce the generation of reactive oxygen species (ROS) due to the presence of iron associated with the fibrous silicates and to the activation of macrophages and inflammation. Chronic inflammation and a ROS-enriched microenvironment can foster the malignant transformation of mesothelial cells. In addition, MPM cells have a highly glycolytic metabolic profile and are positive in ^18^F-FDG PET analysis. Loss-of-function mutations of BRCA-associated protein 1 (BAP1) are a major contributor to the metabolic rewiring of MPM cells. A subset of MPM tumours show loss of the methyladenosine phosphorylase (MTAP) locus, resulting in profound alterations in polyamine metabolism, ATP and methionine salvage pathways, as well as changes in epigenetic control of gene expression. This review provides an overview of the perturbations in metabolism and ROS homoeostasis of MPM cells and the role of these alterations in malignant transformation and tumour progression.

## Background

Malignant pleural mesothelioma (MPM) is a rare malignancy of mesothelial cells that is mainly linked to asbestos exposure and ensuing oxidative stress and chronic inflammation.^1,2^ Asbestos is classified in two major categories, named amphibole and serpentine. There are five types of amphibole named crocidolite, amosite, tremolite, anthophyllite and actinolite, and one type of serpentine, named chrysotile. All types of asbestos fibres have a stretched needle-like shape, but amphiboles are more rigid and have a rod-like morphology, while chrysotile fibres are curly and more flexible. Many studies have demonstrated that amphiboles are highly carcinogenic compared with chrysotile fibres.^1,3^ While most inhaled asbestos fibres are eliminated by mucociliary clearance and alveolar macrophages (AM), the longer fibres (>10–20 µm) cannot be eliminated and result in chronic AM activation and “frustrated phagocytosis”.

Although environmental exposure to asbestos is a major driver of MPM carcinogenesis, recent studies have revealed an important role for germline or somatic mutations in the BRCA-associated protein 1 (BAP1) gene. BAP1 loss-of-function mutations are present in families predisposed to mesothelioma and in 60% of patients with sporadic mesothelioma.^4^ As BAP1 is implicated in many cellular processes, including DNA repair and apoptosis, its mutation may act in co-operation with environmental exposure to asbestos to drive MPM carcinogenesis.^1,2,5,6^ While MPM arising in the context of germline BAP1 mutations occurs in younger patients and is characterised by a better prognosis, sporadic MPM is in most cases refractory to therapy.^7–9^

## Asbestos exposure, ROS and inflammation

Reactive oxygen species (ROS) are a class of chemically reactive molecules derived from the incomplete reduction of molecular oxygen (O_2_). ROS play a pivotal role in asbestos toxicity.^3,10–12^ Reduction of O_2_ by a single electron generates superoxide anion (O_2_^·−^), a membrane-impermeant ROS that is rapidly converted into hydrogen peroxide (H_2_O_2_) by superoxide dismutases (SOD) 1 and 2, localised in the cytosol and mitochondria, respectively.^12,13^ H_2_O_2_ can then be converted into H_2_O by catalases, glutathione peroxidases (GPXs) or thioredoxin peroxidase (TrxP), or reacts with free Fe^2+^ to form the hydroxyl radical (OH^·^) through the Fenton and Haber–Weiss reactions.^13^ O_2_^·–^ can also react with nitric oxide (NO) to produce the reactive nitrogen species peroxynitrite (ONOO^–^).^10,11,13^ High levels of ROS induce macromolecular damage by oxidising proteins, lipids and nucleic acids, which can lead to cell death. On the other hand, physiological levels of ROS may stimulate signal transduction pathways, including the nuclear factor κB (NF-κB), mitogen-activated protein kinase (MAPK) and phosphoinositide 3-kinase (PI3K) signalling cascades.^12,14^

Asbestos fibres increase ROS levels both by a direct mechanism and through induction of frustrated phagocytosis. Undigested asbestos fibres, coated with mucopolysaccharides, iron and iron-containing proteins,^15^ directly induce ROS through the Fenton and Haber–Weiss reactions and maintain a state of local chronic inflammation. The recruitment of phagocytes and activation of myeloperoxidase and vacuolar NADPH oxidase (NOX) results in the production of hypochlorite radicals and ROS, respectively, generating a vicious circle that fuels a highly pro-oxidant inflammatory microenvironment.^1,16–18^ Asbestos fibres also induce the expression of ferritin heavy chain,^19^ which may favour accumulation of iron and thus increase OH^·^ production.^16,17^ Consistent with this notion, the carcinogenicity of asbestos fibres is, at least in part, linked to their iron content and iron oxidation status (Fe^2+^ versus Fe^3+^).^20^ Due to their increased iron content^20^ and rigidity, amphiboles are more carcinogenic than chrysotile fibres. Nevertheless, the content of iron alone is not sufficient to predict the grade of carcinogenicity of asbestos fibres. In this regard, erionite, an asbestos-like silicate structurally similar to amphibole asbestos^21^ but with a lower iron content, shows the highest potency in inducing MPM^22^ probably due to its high biopersistence.^23^ In contrast, palygorskite, a silicate characterised by low biopersistence and low iron content, is not carcinogenic.^24^

Using a mouse model of asbestos-induced peritoneal inflammation, Pietrofesa et al.^25^ demonstrated that injection of crocidolite into the peritoneum induced lipid peroxidation and an increase in total white blood cells in the peritoneal lavage fluid. Peritoneal lavage fluids of crocidolite-treated mice were enriched in pro-inflammatory molecules, including interleukin (IL)-1β, IL-6, high-mobility group box 1 (HMGB1), tumour necrosis factor (TNF)-α and fibrogenic cytokines, such as transforming growth factor (TGF)-β1. The administration of an antioxidant diet, enriched in the flaxseed lignan secoisolariciresinol diglucoside, before asbestos exposure reduced oxidative stress and inflammation in this experimental model. Conversely, the non-carcinogenic silicate mineral fibre palygorskite^2^ failed to induce inflammation when injected into the peritoneum of transgenic mice expressing SV40 TAg in mesothelial cells (MexTAg) and Balb/c mice.^24^

**Fig. 1:**
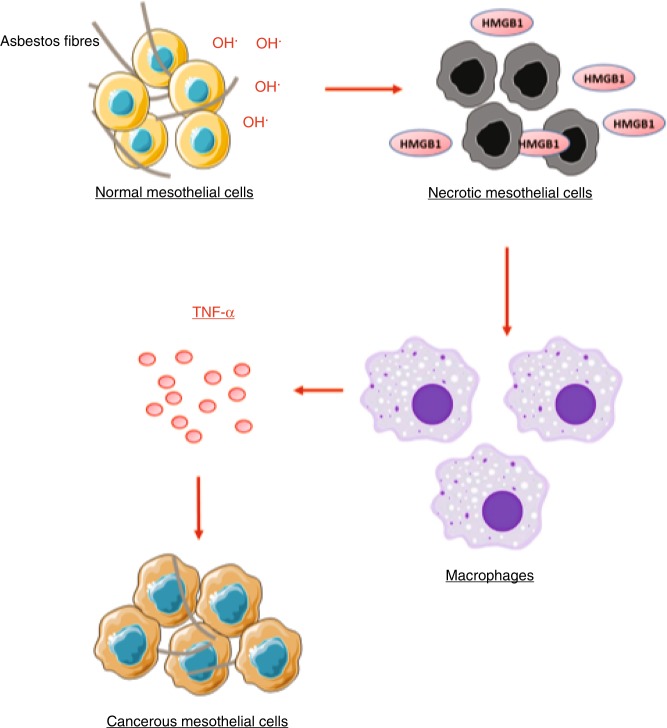
Schematic representation of asbestos-induced mesothelial cell transformation. ROS generated by asbestos fibres induce necrosis of mesothelial cells and release of HMGB1. The latter activates macrophages that in turn secrete TNF-α. TNF-α inhibits the necrosis of mesothelial cells and induces cell proliferation. This and subsequent figures were created using Smart Servier Medical Art (https://smart.servier.com/).

Yang et al.^26^ provided evidence for a mechanism linking asbestos-related ROS production to mesothelium inflammation and cell overgrowth, in a process involving HMGB1 and TNF-α. The authors demonstrated that crocidolite and H_2_O_2_ provoke necrosis of normal mesothelial cells and release of HMGB1 in culture media, which induce TNF-α secretion by macrophages. HMGB1 belongs to the damage-associated molecular pattern (DAMP) family of molecules. In physiological conditions, HMGB1 is localised in the nucleus and exerts its function as a transcriptional cofactor. When cells undergo necrosis, HMGB1 is released in the extracellular space, where it first acts as a chemoattractant for monocytes and then stimulates macrophages to produce pro-inflammatory cytokines, including TNF-α^27^ (Fig. 1).

**Fig. 2:**
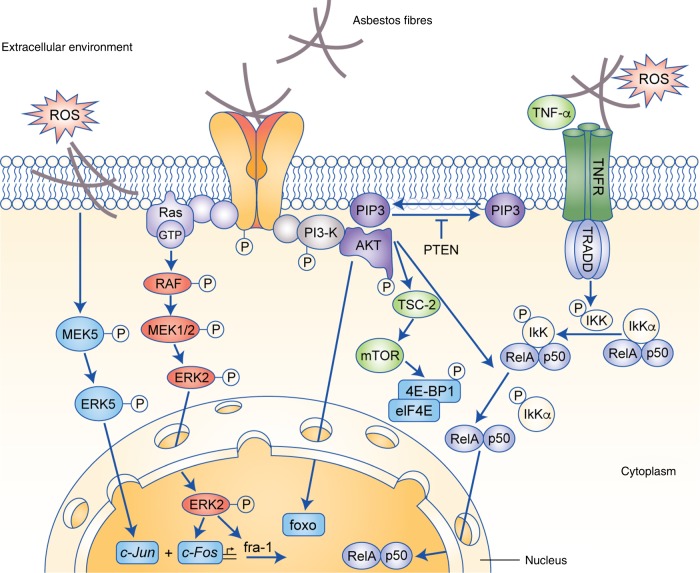
Mitogenic signalling pathways activated by asbestos. Exposure to asbestos leads to the activation of key mitogenic pathways. See main text for definitions.

HMGB1 activity is regulated by the oxidised/reduced status of three cysteine residues (C23, C45 and C106). Its chemoattractant function requires all cysteine residues to be reduced, while a disulfide bond between C23 and C45 favours its binding to Toll-like receptor 4 (TLR4) and induction of cytokine release by macrophages. Completely oxidised HMGB1 seems to be inactive.^28,29^ TNF-α, released by macrophages, inhibite asbestos-induced necrosis and promote mesothelial cell proliferation through activation of the canonical NF-κB (p50/RelA) pathway^30^ (Fig. 2), with consequent enhanced transcription of target genes coding for cytokines, chemokines, adhesion molecules and inhibitors of apoptosis.^31^ Janssen et al.^32^ demonstrated that crocidolite induced NF-κB activation, both in an in vitro model of tracheal epithelial and pleural mesothelial cells, and in vivo in the lungs of rats exposed to crocidolite fibres. Pre-treatment of the rat lung fibroblast cell line RFL-6 with the antioxidant vitamin E prior crocidolite exposure blocked binding of NF-κB to target DNA, suggesting that activation of this pathway by asbestos is ROS-mediated.^33^

## Effects of ROS on TGF-β in MPM

TGF-β is a potent cytokine secreted by several types of inflammatory cells and cancer cells.^34^ TGF-β exists in three different isoforms (TGF-β1, TGF-β2 and TGF-β3), with TGF-β1 being the most abundant isoform in cancer.^35^ TGF-β1 can either suppress or promote tumour growth depending on the tumour stage and its interactions with other components of the microenvironment. TGF-β is secreted as a latent complex in which mature dimeric TGF-β is associated with latency-associated protein (LAP) and latent TGF-β-binding protein (LTBP).^36^ In the extracellular space, matrix metalloproteinases (MMPs), plasmin, thrombospondin-1, binding of integrins or oxidising conditions induce cleavage or a conformational change in LAP that results in the release of active TGF-β^37^ able to bind TGF-β receptor II (TβIIR). This interaction triggers dimerisation of TβIIR with TGF-β receptor I (TβIR) and activation of downstream signalling. This dimerised receptor is a transmembrane serine/threonine kinase that phosphorylates and activates the small mother against decapentaplegic (SMAD) proteins, SMAD2 and SMAD3. Phospho-SMAD2/3 form a complex with SMAD4, move into the nucleus and regulate the expression of hundreds of genes.^38^

In early-stage epithelial cancers, TGF-β acts as a tumour suppressor by provoking cell cycle arrest through induction of the cell cycle inhibitors p15^INK4^, p21^CIP1^ and p27^KIP1^.^39^ However, cancer cells frequently upregulate cyclin-dependent kinases (CDKs), which counteract the effects of these cell cycle inhibitors.^40^ In advanced-stage disease, TGF-β thus acts as a pro-oncogenic factor that promotes tumour invasion, angiogenesis and immune tolerance.^41^

Several studies support a role for TGF-β in MPM biology. MPM cell lines secrete both active and inactive forms of TGF-β in their culture media.^42^ DeLong et al.^43^ demonstrated that pleural effusions obtained from MPM patients contained higher levels of TGF-β compared with pleural effusions derived from patients with breast cancer and non-small-cell lung cancer. Immunohistochemical (IHC) analysis of MPM tissues revealed strong nuclear accumulation of phospho-SMAD2, suggesting constitutive activation of TGF-β signalling in this cancer.^44^ Using an immunocompetent syngeneic mouse model, Suzuki et al.^45,46^ demonstrated that TGF-β has a pro-oncogenic role in MPM that is related to immune suppression. These authors observed strong inhibition of tumour growth in mice treated with soluble TβIIR or SM16, an inhibitor of TβIR. Interestingly, depletion of CD8^+^ T cells using anti-CD8 antibodies reverted the effect of TβIIR inhibition,^45,46^ suggesting that the activity of soluble TβIIR is mediated by CD8^+^ lymphocytes. TGF-β regulates the innate immune system by directing macrophages and neutrophils to acquire an immune-suppressive phenotype. It also stimulates the expression of immunosuppressive T regulatory (Treg) lymphocytes and inhibits proliferation and differentiation of cytotoxic CD8^+^ T cells.^34,41^

TGF-β is also the most important mediator of the epithelial-to-mesenchymal transition (EMT), which confers stem-like features and activates a programme of invasive growth of cancer cells.^47^

During EMT, cells lose the epithelial phenotype and acquire an undifferentiated mesenchymal phenotype.^48^ Despite their mesodermal derivation, mesothelial cells possess some phenotypic features of epithelia, including cell polarity, cytokeratin expression and cell junctions^49^ and undergo EMT when stimulated. Fassina et al.^50^ analysed EMT markers in a large panel of malignant pleural and peritoneal mesothelioma biopsies representing the three histologic subtypes (epithelioid, biphasic and sarcomatoid). Tumours of the most aggressive sarcomatoid subtype showed higher expression of the EMT transcriptional regulators SNAIL, SLUG, TWIST, ZEB1 and ZEB2, and higher expression of the mesenchymal markers vimentin, S100A4, N-cadherin, α-SMA (α-smooth muscle actin) and MMP9 compared with tumours of the biphasic and epithelioid subtypes. On the other hand, epithelial markers including E-cadherin, cytokeratin 5/6 and β-catenin were strongly expressed in epithelioid, weakly in biphasic and absent in sarcomatoid mesothelioma samples.^50^ Taken together, these results suggest that in MPM, TGF-β acts both on MPM and immune cells, supporting tumour growth and aggressiveness.

Crosstalk between ROS and TGF-β has been described, with ROS in the extracellular space capable of inducing activation of TGF-β.^51,52^ Using an in vitro model, Pociask et al.^53^ demonstrated that ROS generated by asbestos-associated iron induced oxidation of LAP and activation of TGF-β1. The addition of antioxidants in the culture media before asbestos exposure reverted this effect, clearly demonstrating the role of ROS in TGF-β1 activation. Nagai et al.^54^ showed that the administration of oral iron chelator, deferasirox, to rats exposed to crocidolite by peritoneal injection did not suppress mesothelioma carcinogenesis but increased the frequency of epithelioid tumours compared with those of the sarcomatoid type, thus supporting a link between iron-associated ROS and EMT. A recent study by Turini et al.^55^ demonstrated that exposure of the SV40 T antigen-immortalised mesothelial cell line MET5A to chrysotile induced EMT in a process mediated by TGF-β1.

TGF-β1 can, in turn, increase mitochondrial ROS through inhibition of the electron transport chain (ETC) complex III or IV, thus favouring electron leak and ROS production by the ETC.^29,56^ TGF-β1 also suppresses antioxidant systems and induces NOX 2 and 4^37^, with subsequent NOX-catalysed production of ROS from O_2_. MPM cell lines express high levels of NOX4.^57^ TGF-β1 was shown to induce the expression of NOX4 in human peritoneal mesothelial cells, while NOX4 knockdown reduced TGF-β1-induced EMT.^58^ However, MPM cells also express high levels of antioxidant systems including catalase, manganese superoxide dismutase (Mn-SOD)^59,60^ and peroxiredoxins (PRX),^61^ which may blunt the impact of high NOX4 levels. Importantly, elevated intracellular ROS potentiate TGF-β-induced EMT by activating NF-κB and HIF (hypoxia-inducible factor),^62^ two transcription factors able to induce the expression of SNAIL, SLUG, TWIST and ZEB1.^63,64^

## Activation of mitogenic signalling pathways

The activation of signalling pathways controlling cell proliferation may be triggered by the direct interaction of mesothelial cells with asbestos fibres or arises as a result of synergism between this direct effect and the chronic inflammatory state induced by asbestos exposure.

In vitro studies demonstrated that the exposure of pleural mesothelial cells to asbestos fibres induces expression of the epidermal growth factor receptor (EGFR) at both the mRNA and protein levels.^65–67^ Crocidolite fibres longer than 20 μm are deposited on the surface of mesothelial cells, where they interact directly with the EGFR, causing its dimerisation and autophosphorylation.^66^ Activation of the EGFR following exposure to asbestos fibres triggers the mitogen-activated protein kinase (MAPK) signalling pathway, which results in the activation of the extracellular signal-regulated kinases (ERKs), c-Jun N-terminal kinases (JNK), stress-activated protein kinase (SAPK) and p38 kinase (p38K) (Fig. 2).

Jimenez et al.^68^ demonstrated that exposure of rat pleural mesothelial cells to crocidolite or H_2_O_2_ induced phosphorylation and activation of ERK, which was prevented by addition of catalase, chelation of surface iron from crocidolite fibres or addition of the ROS scavenger N-acetyl-l-cysteine (NAC), suggesting a causal role for ROS in these changes.

Activator protein-1 (AP-1), a transcription factor composed of c-Jun and c-Fos homo- and hetero-dimers, is known to be sensitive to the redox state of the cell. The phosphorylation of c-Jun and c-Fos by the MAPK signal transduction pathway determines AP-1 activation that in turn induces cell proliferation.^69^ Activation of AP-1 by asbestos fibres was revealed in studies that measured a dose-dependent increase in c-Jun and c-Fos mRNAs in rat pleural mesothelial cells and rat bronchial epithelial cells exposed to crocidolite.^70^ Pretreatment with NAC prevented the asbestos-triggered upregulation of c-Jun and c-Fos.^71,72^ Non-fibrous materials, chemically similar to crocidolite, were not able to alter the expression of c-Jun and c-Fos, indicating that the physical properties of asbestos fibres is essential for this effect.^70^

The overexpression of c-Fos has also been described in the mesothelial cells of rat pleura exposed to crocidolite fibres. This induction, mediated by the activation of EGFR and ERK1/2, was associated with apoptosis.^73^ AP-1 was also induced in pleural mesothelial cells exposed to asbestos fibres and correlated with the ability of the cells to grow in soft agar and to acquire a fibroblast-like phenotype. These changes were mediated by an ERK-dependent increase in the expression of the AP-1 subunit FOSL1 (*fra-1*).^74^

The cell’s redox state also regulates PI3K–AKT and the downstream mammalian target of rapamycin (mTOR) pathway, both of which are critical modulators of cell growth and survival. The PI3K–AKT pathway is often found to be activated in MPM.^75–77^ Signalling of this pathway is inhibited by the phosphatase phosphatase and tensin homolog (PTEN), which dephosphorylates phosphatidylinositol (3,4,5)-trisphosphate (PIP3) into phosphatidylinositol (4,5)-bisphosphate (PIP2). Interestingly, PTEN is inactivated by H_2_O_2_ through oxidation of cysteine residues.^78^

These studies are interesting, but they should be considered with caution as they are based on short-term experiments carried out on MPM cell lines, which died following treatment with asbestos, arguing against a direct carcinogenic effect of asbestos on MPM cells.

## Redox homoeostasis in MPM therapies

Most cancer cells maintain a higher ROS setpoint compared with normal cells due to the selective advantage of ROS-dependent engagement of mitogenic pathways. However, these levels are close to the threshold that triggers cell death.^79,80^ It is therefore not surprising that cancer cells are more vulnerable to ROS-inducing drugs and/or inhibitors of antioxidant pathways than their normal counterparts. Consistent with this notion, Denis et al.^81^ showed that treatment of MPM cell lines with the experimental antitumour agent phenethyl isothiocyanate (PEITC) induced ROS production and potentiated cisplatin-induced cell death without producing any effect in normal mesothelial cells.^81^ Several studies demonstrated the potential utility of ROS-inducing therapies for mesothelioma treatment.^82^ However, overexpression of antioxidant systems may explain the resistance of MPM tumours to ROS-generating therapies such as cisplatin/pemetrexed, epirubicin and radiotherapy.^81–84^ First-line platinum-pemetrexed chemotherapy represents the current standard of care for advanced MPM treatment. Chen et al.^85^ demonstrated that cisplatin, which is known to kill cancer cells primarily through induction of DNA damage, also increased mitochondrial ROS in MPM cell lines. Greater ROS accumulation was related to higher sensitivity to cisplatin, and this effect was abrogated by treatment with the ROS scavenger NAC, while knockdown of glutathione-S-transferase enhanced this effect. Nuvoli et al.^86^ showed that the sensitivity of MPM cell lines to death induced by the aromatase inhibitor exemestane correlated with increasing intracellular ROS, ERK phosphorylation and a decrease in signal transducer and activator of transcription 3 (STAT-3) phosphorylation; these effects were abrogated when the cells were pre-treated with NAC. Of note, one MPM cell line that was resistant to exemestane exhibited a treatment-dependent activation of nuclear factor erythroid 2-related factor 2 (Nrf2), a transcription factor implicated in the oxidative stress response, and in increased expression of the antioxidant enzymes catalase, SOD and GPX, as well as γ-glutamylcysteine (a precursor of glutathione). Cunniff et al.^87^ showed that thiostrepton and triphenylmethane gentian violet, inhibitors of PRX3 and TRX2, respectively, inhibited tumour growth in an in vivo MPM mouse model.

These studies provide a strong rationale to simultaneously target ROS-producing and ROS-scavenging pathways to selectively kill MPM cells.

## Metabolic rewiring of MPM cells

The metabolic rewiring of cancer cells towards a glycolytic (Warburg) profile is a well-established hallmark of the transformed phenotype,^88^ and MPM cells are not an exception to this rule. In fact, MPM lesions are commonly highly glycolytic and positive in positron emission tomography with 2-deoxy-2-[fluorine-18]fluoro-D-glucose (^18^F-FDG PET) analysis, which is performed for the clinical assessment and follow-up of these patients.^89–91^ Several pathways contribute to favour the Warburg phenotype, including those controlled by PI3K–AKT, HIF, p53, MYC and AMPK (AMP-activated protein kinase).^92^

The transcription factor HIF is a pivotal regulator of the glycolytic phenotype in cancer cells. In the presence of O_2_, the hydroxylation of proline residues 402 and 564 of the HIF subunit HIF-1α enables the tumour-suppressor protein von Hippel–Lindau (VHL) to ubiquitinate HIF-1α, leading to its proteasomal degradation. O_2_ deficiency, such as the hypoxic conditions found in tumour masses, results in HIF-1α accumulation, dimerisation with HIF-1β to form the HIF-1 transcription factor and activation of gene transcription.^93^ HIF-1 promotes tumour angiogenesis by inducing vascular endothelial growth factor and its receptors (VEGF and VEGFRs), and causes a shift towards a glycolytic phenotype by inducing the transcription of the genes coding for the glucose transporters (GLUT1 and GLUT3), pyruvate dehydrogenase kinase 1 (PDK1) and lactate dehydrogenase A (LDHA).^94^ The resulting glycolytic profile allows  cancer cells to generate ATP in hypoxic conditions and allows the diversion of carbons from catabolic processes to macromolecule biosynthesis and redox status maintenance.^92^ Moreover, the metabolic rewiring of cancer cells is intertwined with EMT in a two-way interaction. Indeed, EMT programme requires metabolic changes, achieved through the expression of key metabolic enzymes that are regulated by EMT transcription factors.^95^

ROS can increase HIF-1α transcription and translation through the PI3K–AKT–mTOR pathway and can induce, also in normoxic conditions, HIF-1α stabilisation through inhibition of prolyl-4-hydroxylases (PHD).^96^ The PI3K–AKT pathway is often activated in mesothelioma^75–77^ probably because of ROS-mediated inactivation of PTEN through the oxidation of its cysteine residues.^78,96,97^ A link between the PI3K–AKT–mTOR pathway and metabolic rewiring of mesothelioma cells has been suggested by Kaira et al.^98^, who reported a positive correlation between ^18^F-FDG uptake and expression levels of HIF-1α, GLUT1, hexokinase, VEGF and phospho-mTOR in a small cohort of MPM patients.^98^ Moreover, the authors demonstrated that inhibition of both HIF-1α and mTOR decreased ^18^F-FDG uptake in MPM cell lines.

**Fig. 3:**
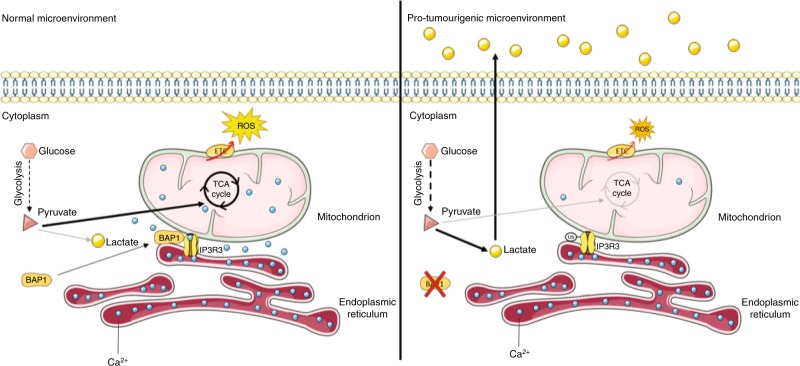
Metabolic rewiring and ROS production controlled by BAP1. (Left) BAP1 deubiquitylates and activates IP3R3, thus increasing the concentration of calcium in mitochondria, which promotes the activity of TCA cycle. The consequent enhancement of electron flow (red arrow) through the electron transport chain (ETC) also drives ROS production. (Right) When BAP1 expression is lost or reduced, the ubiquitination (Ub) of IP3R3 impairs its activity, reducing calcium levels in mitochondria. This reduces the activity of the TCA cycle and electron flow across the ETC, thus decreasing ROS production and favouring a switch to glycolytic metabolism (Warburg effect). Lactate excreted in the microenvironment generates a pro-tumorigenic environment.

Furthermore, loss-of-function mutations in the BAP1 gene profoundly affect the levels of metabolites connected to glycolysis and tricarboxylic acid  (TCA) cycle,^99^ with a reduction in mitochondrial respiration and an increase in glucose consumption and lactate production.^100^ These alterations lead to acidification of the tumour microenvironment and promote the polarisation of tumour-associated macrophages towards a M2 phenotype, resulting in an immune-suppressive milieu that favours malignant transformation.^100^ Thus, through its effects on the metabolic profile of mesothelial cells, loss of BAP1 promotes a favourable niche for malignant transformation and tumour progression.^101^ The mechanisms through which loss of function of BAP1 promotes the Warburg effect are not completely understood. Bononi et al.^99^ observed that BAP1 localises to the endoplasmic reticulum, where it deubiquitylates type-3 inositol-(1,4,5)-trisphosphate receptor (IP3R3), resulting in increased release of Ca^2+^ from the ER, which may lead to increased Ca^2+^ uptake by mitochondria. Although calcium overload in mitochondria is associated with the induction of permeability transition pore-mediated cell death,^102^ moderate increases of intramitochondrial calcium stimulate the activity of enzymes of the  TCA cycle,^103^ thus promoting mitochondrial respiration. Consistent with this notion, low/absent BAP1 activity is associated with decreased activity of the ETC, resulting in a more glycolytic, “Warburg-like” metabolic profile (Fig. 3).

Interestingly, Hebert et al.^104^ also showed that BAP1 increases the intracellular level of ROS in mesothelioma cells, and that treatment with the antioxidant NAC inhibits the effects of BAP1 in the regulation of cell morphology, cell migration and mitochondrial respiration.^104^

The metabolic profile of MPM cells influences their sensitivity to the standard chemotherapy protocols based on the association of platinum-derived compounds with antimetabolite drugs (e.g. pemetrexed). A recent study^105^ investigated the basis for the acquired resistance to cisplatin using the MPM cell line P31 as a model. Interestingly, cisplatin-resistant cells generated by drug-induced selective pressure in vitro exhibited altered redox homoeostasis with increased ROS levels.

The acquired cisplatin resistance was associated with profoundly altered bioenergetic features of the cells, including a decrease in the oxygen consumption rate, an alteration that was accompanied by a reduction in mitochondrial content and decreased expression of proteins involved in mitochondrial biogenesis. Interestingly, the expression of HIF-1α protein was increased in cisplatin-resistant cells, which is consistent with the central role of HIF-1 in driving the metabolic rewiring of cancer cells towards a more glycolytic phenotype^105^ and with the key role of ^18^F-FDG PET in predicting the response of MPM patients to chemotherapy.^89,90^

In vitro studies investigated the possible connection between the response to pemetrexed and the expression of enzymes targeted by the drug (e.g. thymidylate synthase [TS], dihydrofolate reductase [DHFR] and glycinamide ribonucleotide formyltransferase [GARFT]).^106,107^ The data suggested that higher expression levels of DHFR, TS and GARFT correlated with increased sensitivity to pemetrexed, while acquired resistance to the drug seemed to correlate with either increased expression of TS and GARFT or activation of AMPK.^107,108^ Conversely, retrospective studies on MPM patients showed that low TS expression correlated with better response to cisplatin plus pemetrexed therapy.^109^ One must also consider that the response to pemetrexed may be affected by a variety of factors including the uptake and accumulation of the drug, as well as by the addiction of cancer cells to folate cycle-mediated pyrimidine synthesis.^110,111^ Further studies are needed to understand the mechanisms of pemetrexed resistance in MPM and to identify predictive biomarkers to better stratify the patients.

**Fig. 4:**
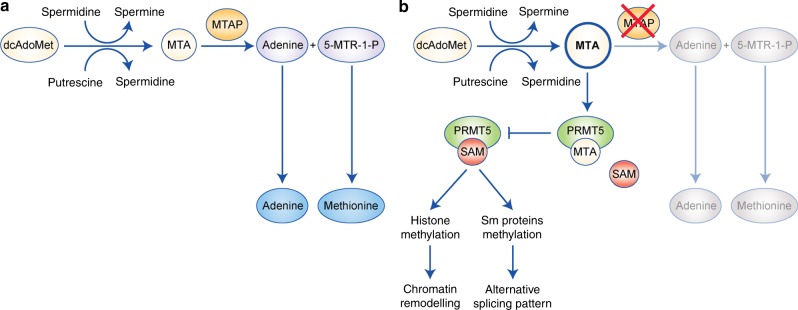
Schematic diagram of the MTAP pathway.** a**. 5′-methylthioadenosine phosphorylase (MTAP) converts MTA into adenine and 5-methylthioribose-1-phosphate (5-MTR-1-P). **b**. Loss of MTAP results in accumulation of MTA, which inhibits protein arginine transferase 5 (PRMT5), an enzyme involved in the methylation of Sm proteins and histones.

Another interesting metabolic alteration is found in a subset of MPM patients carrying the deletion of the methylthioadenosine phosphorylase (MTAP) gene, which is adjacent to the CDKN2A locus, a frequent target of deletion in MPM.^112^ MTAP catalyses the conversion of methylthioadenosine (MTA) into adenine and 5-methylthioribose-1-phosphate, which can be used to make ATP and methionine, respectively. As MTA is derived from a reaction coupled to the conversion of spermidine into spermine and putrescine into spermidine, this pathway links polyamine metabolism to the salvage pathways of adenine and methionine^113^ (Fig. 4). Although inhibitors of purine biosynthesis are predicted to be synthetically lethal in cancers with defects in MTAP, at least in vitro, this effect may be bypassed if tumour cells take up adenine from the microenvironment.^114^ Loss of MTAP also results in accumulation of its substrate MTA, which inhibits protein arginine methyltransferase 5 (PRMT5), a key enzyme that methylates arginine side chains of proteins including histones, thus affecting chromatin remodelling and epigenetic control of gene expression (Fig. 4). Consistent with this notion, MTAP-defective cells are selectively vulnerable to pharmacological inhibition of PRMT5.^115–117^ It will be interesting to assess whether loss of MTAP could be a predictive marker of response of MPM patients to PRMT inhibitors.

In addition, PRMT5 is also involved in the methylation of Sm proteins (Fig. 4), which are involved in mRNA processing,^118–120^ suggesting that PRMT5 inhibitors may also produce major changes in the pattern of mRNA expression.

Interestingly, intraperitoneal injections of asbestos in Nf2^+ /−^ mice result in the development of mesothelioma that recapitulate many of the molecular features of human MPM, including deletions of the CDKN2A locus that span nearby loci.^121^ A significant fraction of these tumours also exhibit loss of MTAP expression.^122^

Another potentially interesting alteration observed in MPM patients is the epigenetic silencing of argininosuccinate synthetase 1 (ASS1), a key enzyme in arginine biosynthesis.^123^ Loss of ASS1 makes MPM cells dependent on exogenous arginine, a metabolic hallmark that may be exploited therapeutically. This possibility is supported by a recent study showing that pegylated arginine deiminase (ADI PEG20), an enzyme that destroys circulating arginine, produced a modest but significant increase in progression-free survival in patients with ASS1-deficient advanced MPM.^123–126^

## Conclusions and perspectives

MPM is an aggressive neoplasm that is tightly linked to the chronic inflammation and oxidative stress triggered by inhaled asbestos fibres. Although key aspects of the aetiopathogenesis of this neoplasm have been elucidated, the prognosis is still dismal for most patients with sporadic MPM. Robust biomarkers for early diagnosis and novel targeted pharmacological approaches are the key unmet clinical needs in MPM management.

Studies discussed in this review describe profound changes in the redox homoeostasis and metabolic profile of MPM cells, providing groundwork for the development of novel diagnostic and therapeutic tools.

Among these, pharmacological approaches aimed at tipping the ROS rheostat to reach cytotoxic ROS levels appear to be particularly attractive to selectively target MPM cells. The direct effects of asbestos fibres, as well as the phagocyte oxidases engaged through chronic inflammation, generate a highly pro-oxidant microenvironment in MPM; it is thus likely that inhibition of the fuelling of scavenging pathways would be the most effective strategy to target this neoplasm by exploiting its redox imbalance.

The ability of asbestos to increase ROS also provides a mechanistic rationale to the carcinogenic “field effect” of these mineral fibres, which results in the formation of multiple foci of atypical mesothelial hyperplasia and is consistent with the observation that mesothelioma is a polyclonal malignancy.^127^ The field effects of asbestos and the polyclonality of MPM has profound implications for therapy as the different clonal MPM populations may carry a distinct set of molecular alterations.^2^ This latter point is of particular interest as recent studies suggest that MPM relapses may arise from novel, clonally distinct, malignancies.^127^

The glycolytic profile of MPM cells, which is particularly prominent in tumours resistant to cisplatin/pemetrexed, makes these tumours potentially vulnerable to compounds that inhibit HIF-1α, or components of the metabolic hubs controlling the fate of pyruvate (e.g. LDHA, PDH and PDK). MPM with loss-of-function mutations of BAP1 may be selectively targeted using inhibitors of histone deacetylases (HDAC), histone methyltransferase (histone–lysine–N-methyltransferase enzyme: EZH2), poly ADP ribose polymerase (PARP) or ribonucleotide reductase regulatory subunit M1.^2^

Loss of MTAP is also interesting, as it potentially generates a condition of synthetic lethality. Genetic (or epigenetic) alterations in this gene are likely to serve as useful predictive markers to stratify MPM patients with the aim of defining a subset that would be eligible for treatments with inhibitors of PRMT5 or other methyltransferases that may be affected by the accumulation of MTA.

The validation of these findings in large-perspective cohort studies will permit identification of the most robust biomarkers as well as the most effective novel druggable targets.

## Data Availability

Not applicable.
